# New Insight on Archaeological Metal Finds, Nails and Lead Sheathings of the Punic Ship from Battle of the Egadi Islands

**DOI:** 10.3390/molecules28041968

**Published:** 2023-02-19

**Authors:** Francesco Armetta, Rosina Celeste Ponterio, Ivana Pibiri, Maria Luisa Saladino

**Affiliations:** 1 Department Biological, Chemical and Pharmaceutical Science and Tecnology, University of Palermo Viale delle Scienze Bld. 17, 90128 Palermo, Italy; 2 CNR—Istitute for Chemical Physical Processes, Viale Ferdinando Stagno D’Alcontres 37, 98158 Messina, Italy

**Keywords:** copper, lead, underwater degradation, sulphides formation, archaeometallurgy, Punic ship

## Abstract

The wreck of the Punic ship exhibited at the Archaeological Park of Lilybaeum (Marsala, Italy) is a unique example in the world. In this paper, the investigation of some metal finds (30 nails and 3 fragments of sheathings) belonging to the wreck of the Punic ship is reported. Portable X-ray fluorescence and Raman spectroscopy allowed us to identify the elements and compounds constituting them and make some deductions about their composition. X-ray diffractometry, polarised optical microscopy and scanning electron microscopy of the collected micro-samples allowed us to explain the degradation that occurred in the underwater environment.

## 1. Introduction

Underwater cultural heritage represents the long-term remains of ancient and modern civilisations. Limestone, ceramics and metals are the most commonly found artefacts; woods are found only in particular environmental conditions. In all cases, their degradation is strongly influenced by the chemical conditions of the surrounding water and burial in sediments. When the objects are encapsulated within well-defined environmental conditions for an inferable time scale, their investigation may serve as a unique laboratory to explore chemical processes through time and for reconstructing the micro-environmental conditions that might have caused observed alterations in their chemical and mechanical properties [1,2,3].

Metals cover a significant percentage of the literature case studies of the several processes involving the specific metal mineralisation and corrosion processes [4,5]. In fact, for most of the metals and their alloys, the environmental oxidant conditions involve a series of electrochemical reactions forming minerals both on the surface and in the bulk of the objects, even if in a few cases the metal is recovered in good condition without significant traces of corrosion phenomena [6]. One of the most common processes regarding copper-based alloys is the so-called *bronze cancer*, which involves cyclic reactions slowly proceeding through chlorine ions [7,8,9,10]. In anaerobic conditions, the bronze can be totally converted into copper and tin sulphides and sulphates due to the presence of sulphate-reducing bacteria [11]. Iron-based metals follow different paths for the formation of corrosion products [12,13,14]. Several corrosion products and processes have been observed in lead-based metals, whose development is influenced by environmental conditions [15]. The analysis of the corrosion products or of the survived metals involves the use of several techniques with a multidisciplinary approach to extract the maximum information available, draw a picture of the conservation state of the object and understand the processes which took place [6,16].

Due to this observed variability, due to the complexity of the ongoing processes, which cannot be predicted, sometimes it is impossible to draw explicit conclusions on the reasons for the present condition of the objects. Therefore, the investigation of archaeological metals is always necessary, especially when they are found in specific and unique archaeological contexts. This is true in the case reported here about the metal nails and sheathings of a Punic ship saved at the Archaeological Park Lilybaeum [17] and recognised as a milestone in the history of archaeology [18,19]. Some details about its discovery are reported in the next paragraph.

## 2. The Punic Ship and the Metal Finds

The Punic ship was discovered in 1971, by chance, off the west coast of Sicily during underwater archaeological surveys by an international team led by Honor Frost. Details and videos about the wreck recovery are reported on the website of the Honor Frost Foundation [18]. Over the course of the following seven years, the remains of the vessel were excavated, recovered, conserved and reconstructed. Miss Frost hypothesised that it was a military long-ship that came to grief following the sea battle between the Romans and Carthaginians that ended the First Punic War on 10 March 241 BC. 

Many studies have been performed on the Punic ship. Some of them are related to the archaeological discovery [20,21,22,23], the conservation of the ship [24,25] or the investigation of the finds of cargo [26]. Only recently, a 3D scan was developed in order to define its conservation state at present [27]. Few studies about metal finds were reported [23,26]. X-ray computed tomography of a fragment shows two nails whose heads seem corroded. They were holding the lead-sheet protection of the hull rather than having a role in the carpentry structure.

**Figure 1:**
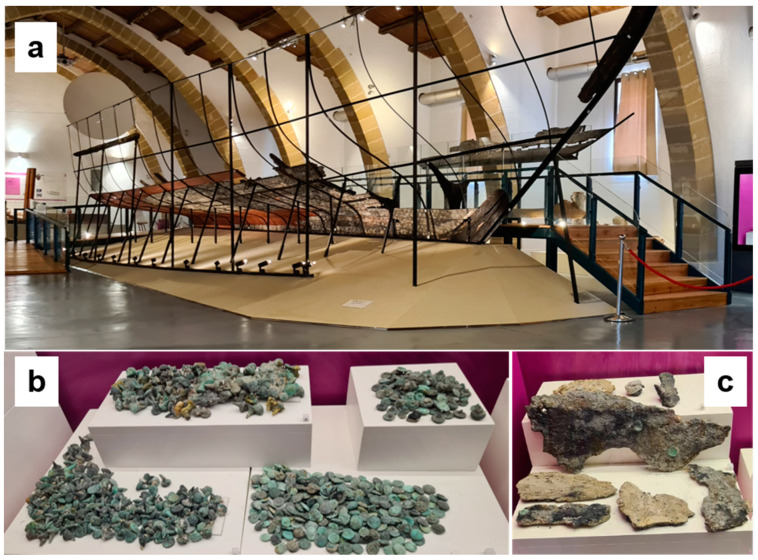
(**a**) Photo of the wreck of the Punic ship exhibited at the Archaeological Park Lilybaeum where nails are inserted in the wood; (**b**) nails and (**c**) sheathings in the showcases.

The planks of the wreck still contain the pattern of nails penetrated on the wood, with the heads still clearly visible (Figure 1a), but a huge group of nails were also found close to the wreck in the same archaeological contexts (Figure 1b) together with blackish fragments of the hull sheaths (Figure 1c). They are very important elements for understanding the ship’s construction method. The batch of finds consists of lead sheets used for the hull lining below the waterline and of several countless pegs and residual heads used for fixing the above sheets, long and folded nails used to ensure the frames of the hull to the planking, wooden dowels with nails inside them and concretions of iron nails. One of the questions asked by the archaeologists themselves at the time of the exhibition concerned the composition of the alloys. In the literature, the nails are defined *tout court* as “copper nails”, but the detailed excavation report, published by H. Frost, addresses the question in a critical and scientific way, deserving further investigation [23]. The English archaeologist reports that the understanding of the alloy nature constituting the nails had involved five years of studies and seventeen tests in three different laboratories. Finally, thanks to the investigation of a nail from the sister ship, which retained a solid metal core inside a thick corrosion layer, it was possible to deduce that the alloy was a bronze, due to the presence of tin, claiming that the tin was “volatilized” from the outer layers due to the marine environment. H. Frost reported the conclusions of Mr Jone, responsible for the laboratory: *“Careful examination of the corrosion product layers surrounding the bronze core of the Sister Ship large nails show that, in the conditions pertaining on this site, tin is readily removed during the corrosion process, consequently such corrosion products cannot be relied on to identify the original material…all the reports that the nails were originally copper must be altered to ‘copper or bronze’”* [23]. The question would have remained unsolved if, as H. Frost ironically claims, Merciful Providence had not come to their aid: during the typological classification of the nails, a nail with a metal core from the Punic ship was identified. The results of the analysis clarified the presence of 7.1% tin, compared to 80% copper and 12.3% lead. These results changed the perspective and in the next reports, the term *“no metal present, corrosion products of copper”* was used.

Today, both nails and sheaths have lost the metallic features and look covered by macroscopically heterogeneous materials suggesting that a strong degradation occurred. In addition, the nails show a large variability of thickness and colours of patina and concretions (Figures S1–S4 in Supporting Information, SI). The determination of the composition can provide helpful information for archaeologists about the materials and the technology of the Punic culture, i.e., one of the questions raised by archaeologists regards the kind of metal used for the production of the nails, there being some doubts about pure copper or copper-based alloys. Considering their long permanence in the underwater environment, the study of their composition is of great interest to the research community involved in underwater degradation [3,28,29,30], but also from the conservation point of view in terms of knowing the process involved in their degradation and planning the better conservation strategy. 

**Figure 2:**
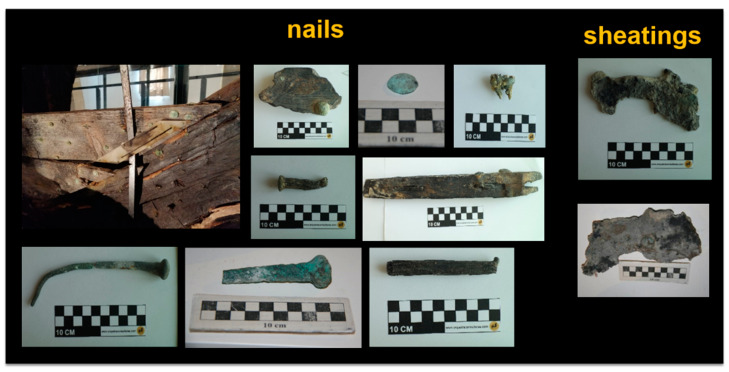
Photos of some investigated nails and sheathings.

In this study, 30 nails and 3 fragments of sheathings were investigated to determine their microchemical composition and to define the degradation processes that occurred in 2000 years of underwater ageing in the Mediterranean seabed. Some of the investigated metals are reported in Figure 2.

## 3. Results and Discussion

The investigation was carried out in two different steps. In the first, portable X-ray fluorescence (*p*XRF) and Raman spectrometer were applied in situ with portable instruments to non-invasively investigate the elemental and molecular composition, respectively. On the basis of the obtained results, a deeper investigation was undertaken through digital microscopy, polarised optical microscopy (POM) and scanning electron microscopy coupled with an energy-dispersion spectrometer (SEM-EDS) by observing three cross-section micro-samples collected from the tip and the head of a nail fragment and from a sheathing. The results of optical microscopy drove a microsampling to perform *p*XRF and X-ray diffractometry (XRD) investigations on powder coming from different layers to better discriminate their composition. The nail tip was sampled by separately collecting the inner and outside parts. Concerning the lead sheathing, small amounts of powders were collected from the two identified layers (black and white). 

### 3.1. Nails

**Figure 3:**
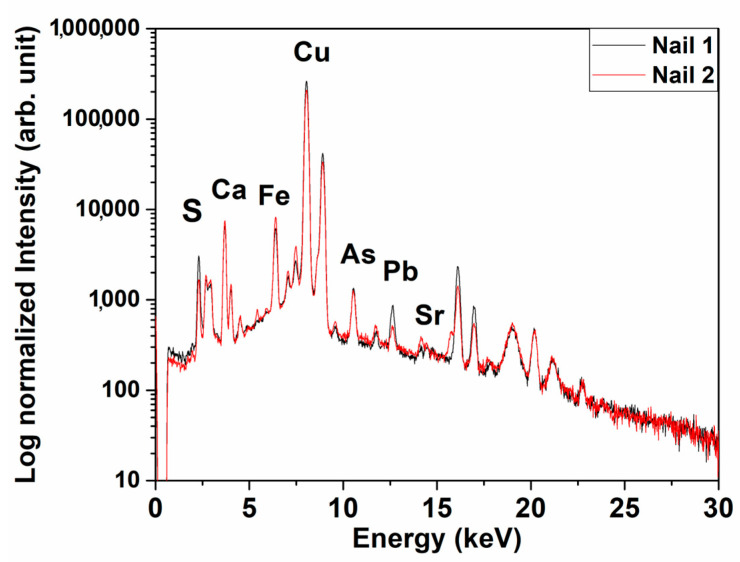
Two representative *p*XRF spectra of the nails.

The ***p***XRF spectra of all analysed spots of the nails are similar (Figure 3). For all nails, the presence of high copper (Cu) peaks was observed together with small peaks of iron (Fe), arsenic (As), lead (Pb), calcium (Ca), strontium (Sr) and sulphur (S). The comparison of the net area of the peaks does not show any significant difference (Table S1 of SI). Cu, As and Pb are related to the metal composition. No signal of tin (Sn) was observed, so it is reasonable to exclude that nails were made of bronze. The presence of Ca and Sr could be due to sea organism source contaminations, whereas the S presence is ascribable to the corrosion pathway, an element characteristic of anaerobic degradation that sometimes occurs in an underwater environment [31]. The Fe presence is due to environmental contamination and could be originated from the surrounding corroded iron objects. The produced ions have been incorporated into the patina or into the external surface, as shown in Figure S2.

Raman spectra show the presence of minerals such as quartz and sulphates on the surface (Figure S5 of SI). In order to get more information about the corrosion processes, the investigation proceeded with the analysis of the collected micro-samples.

**Figure 4:**
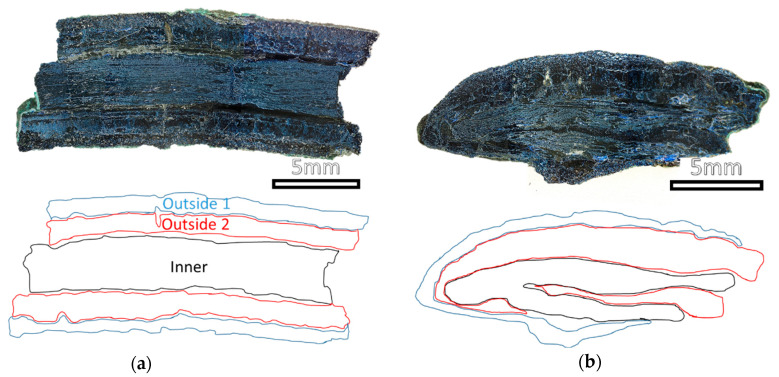
Images acquired with the USB digital microscope and hand draw scheme of the cross-sections of (**a**) tip and (**b**) head of the nail. The layers were drawn by following the borders of the area with different aspects; the same colour was used for areas with similar characteristics in the two samples.

**Figure 5:**
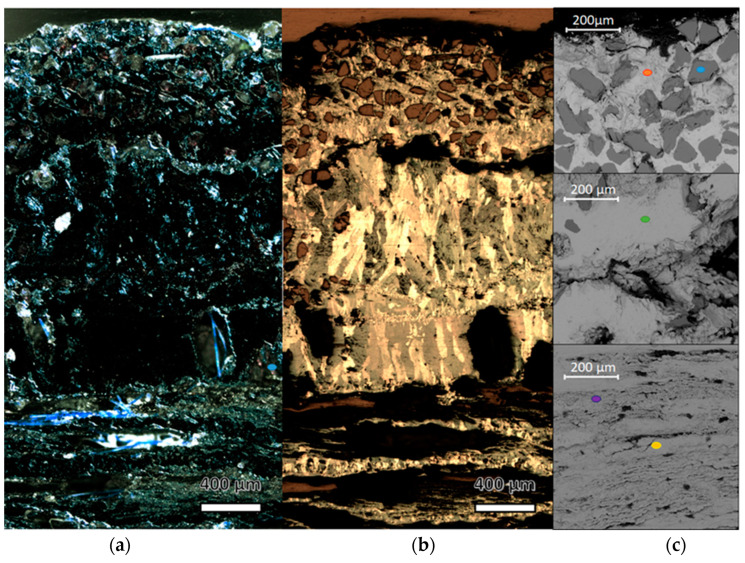
Images acquired from an optical microscope in (**a**) polarised light and (**b**) bright field; (**c**) SEM micrographs of the nail head cross-section. The coloured points indicate the spots analysed by EDS and the relative spectrum is reported in Figure S6 of SI.

Representative micrographs of the cross-sections of a nail are reported in Figure 4 and Figure 5. The images acquired from the cross-sections clearly show the absence of the metal in the inner part, indicating that the mineralisation completely occurred. In the case of the tip, it is possible to observe the presence of layers with different structures (called outside 1, outside 2 and inner). Similar minerals with different distributions can be observed in the head of the nail. The magnifications of the head cross section obtained by POM (Figure 5a,b) help in the morphological discrimination of the layers. In the external layer (outside 1) (Figure 5c), regular polyhedral grains are observed and attributed to quartz grains thanks to SEM-EDS analysis which revealed an elemental composition of Si and O (Figure S6). Below this layer, a dark compact mineralisation stretching for about 1200 μm is observed, where Cu and S are present (Figure S6).

**Figure 6:**
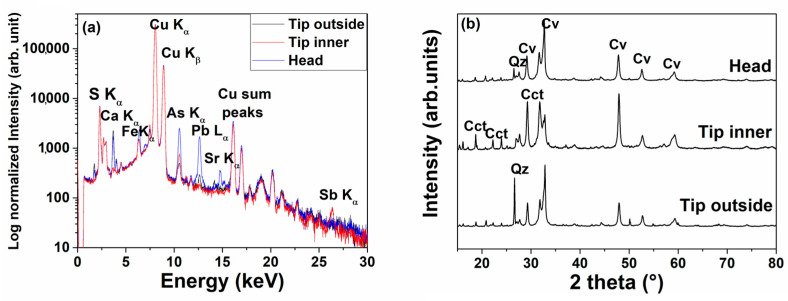
*p*XRF spectra (**a**) (the non-indexed peaks come from instrumental contribution or from elements already indexed) and XRD patterns (**b**) of the powdered nail layers (Cv = covellite, Cct = chalcanthite, Qz = quartz).

The ***p***XRF spectra of the powders (Figure 6a) are in agreement with the ones acquired on the surface with the exception of the inner tip, where a small signal of antimony (Sb) is also recognised. Cu, Fe and As are elements already present in the alloy together with Sb, which was preserved by the deep mineralisation [32]. It is also interesting to note that lead (Pb) is mostly in the head sample, probably due to the interaction with the lead sheathing. The low intensity of the sulphur peak (S) does not indicate a small amount of this element, as its fluorescence efficiency is much lower than those of metallic elements. The comparison of the nails’ ***p***XRF spectra with the spectrum of laboratory copper (II) sulphate highlights a strong similarity in the peak intensity ratio between copper and sulphur, suggesting a similar stoichiometry. 

The presence of quartz in the XRD patterns (Figure 6b), mostly outside the tip, is in agreement with the diffuse presence of sand grains. Together with quartz, the *covellite* (CuS) is recognised and attributed to the compact black mineralisation below the sand layer. The inner part contains both *covellite* and *chalcanthite* (CuSO_4_ 5H_2_O) explaining the observed inhomogeneity with black and blue minerals. Chalcanthite is a secondary mineral that is formed in the oxidation zone of copper sulphide deposits. The head of the nail contains all the phases observed in the two parts of the tip.

The composition of the nails provides information related to the kind of metals, the production processes and the corrosion involved during underwater ageing. The simultaneous presence of arsenic and antimony traces suggests the use of not completely roasted sulphide ores (i.e., *enargite* Cu_3_AsS_4_) and/or rather poor refining practices [33]. This finding related to the metallurgy of nails is important because it can be an indication of the use of copper sulphide ores as a mineral source [34]. The identification of *covellite* and *chalcanthite* clearly indicates that the mineralisation of the metal occurred in anaerobic conditions, where sulphide ions drove the formation of these corrosion products. Usually, copper in an aerobic environment such as seawater is oxidised quite rapidly [35] on a scale of a few tens of microns per year of *atacamite* (Cu_2_(OH)_3_Cl), often with a mixture of cupric hydroxide and basic cupric carbonate. This corrosion layer is toxic to microorganisms and protects the copper from sulphides. Therefore, the sulphidization of the copper nails could have occurred only if they had reached anaerobic conditions when they were relatively new, or if their unexposed surfaces were close to a rich bacterial environment.

Lead was not detected in significant amounts for all the nails and even in the inner layer of the nail tip suggesting that it was not added during the copper casting. 

The manufacturing was usually performed by a hammering process [19] because lead is not soluble in copper alloys, as when the temperature drops to the ambient one, dendrites clearly separate from the copper phase form, and these lead islands penalise the mechanical resistance and handwork production, as suggested by Griesser [36] and Di Turo [37], so the absence in the nail is reasonable considering the required mechanical properties for this kind of object. The presence of a small amount of lead in the head of the nail could be due to the diffusion of lead ions from the lead sheathing which it was in contact with.

### 3.2. Sheathings

**Figure 7:**
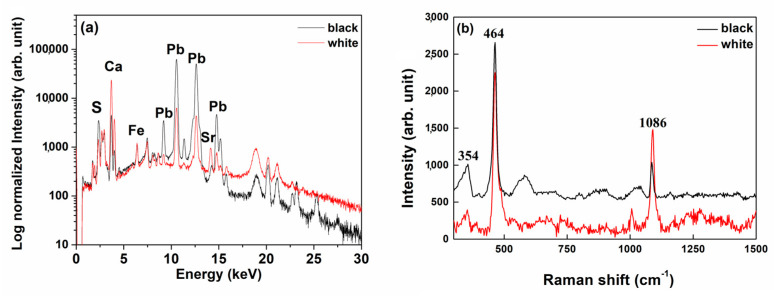
Two representatives (**a**) *p*XRF and (**b**) Raman spectra of the sheathings.

All ***p***XRF spectra of the sheathings show the characteristic fluorescence L lines of Pb, with few differences between the black and white areas. The surface white area is characterised by a high Ca signal, while the inner blackish area contains S peaks (Figure 7a). Raman spectra (Figure 7b) show the signal at 464 and at 1086 cm^−1^ characteristic of the quartz and calcite, respectively. Furthermore, a small signal between 975 and 1040 cm^−1^ is assigned to the sulphate group and the presence of broad bands is probably due to lead sulphide in the spectrum of the black area.

**Figure 8:**
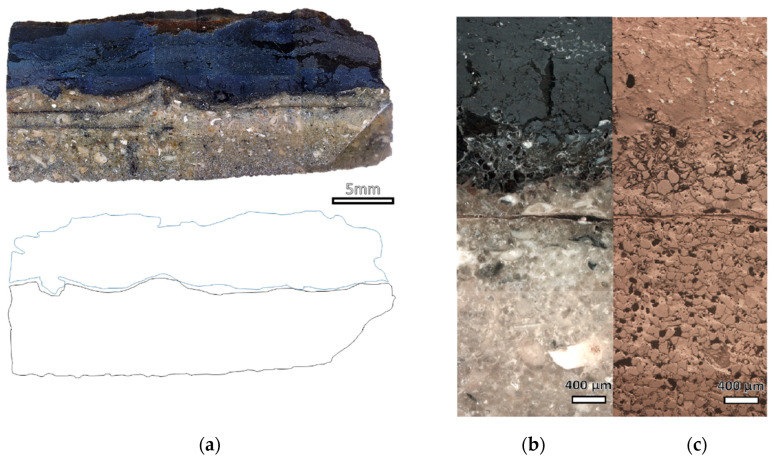
Images acquired from a digital microscope and hand draw scheme of the cross-sections of the lead sheathing (**a**), magnification of the boundary between the two layers at optical microscope under polarised light (**b**) and bright field (**c**).

The optical microscope images of the sheathing of the cross-sections show the complete mineralisation of the metal (Figure 8a). It is possible to observe that the white and black areas are constituted by two layers with similar thicknesses. The magnification with dark and bright fields describes the microchemical structure of the minerals constituting the two layers (Figure 8b). While the black layer is constituted by mineralisation products of the lead, the white layer is composed of quartz grains of similar size (seen from the light bright micrography in Figure 8c) of the ones observed for nails but with a more compact packing and with a white binder among grains—the calcite identified with the Raman investigation. There is only a small connecting interface between the two layers (~800 μm) where it is possible to observe the lead quartz grains embedded into the black minerals.

**Figure 9:**
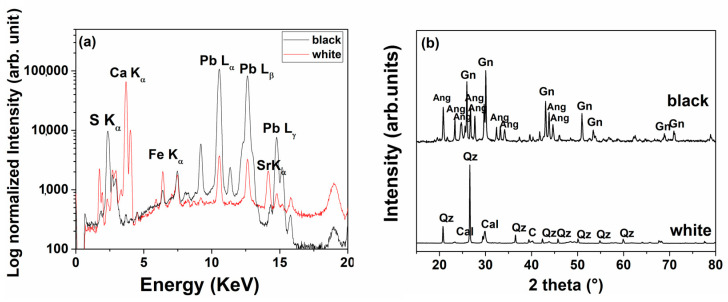
*p*XRF spectra (**a**) and XRD patterns (**b**) (Gn = galena, Ang = anglesite, Cal = calcite, Qz = quartz) of the powdered layers.

The ***p***XRF spectra of the powders (Figure 9a) are analogous to the previous one obtained on the surface of the two sides with the exception of the calcium content, in fact, the signals of calcium are almost absent in the case of the powder from the black layer. Effectively, in the black layer, there is no evidence of the presence of white crystals and probably the calcium signal identified on the surface comes from an environmental deposit on the surface. The XRD pattern of the black part of the sheathing (Figure 9b) shows the peaks of galena (PbS, Ga) and anglesite (PbSO_4_, A), while the pattern of the white part is mainly constituted by quartz (Q) and calcite (C) peaks.

The mineralisation of lead into lead sulphides and sulphates is another proof of the occurrence of anaerobic conditions [15].

It was interesting to observe that one side of the lead sheathing is covered by a thick layer of sand concretion, a possible indication that this side was in full contact with the seabed while the other side was attached to the wood of the shipwreck. The formation of millimetre concretion layers during underwater ageing is common for lead finds and are usually composed of a mixture of marine seabed debris (i.e., sand) and anglesite (PbSO_4_) and calcite (CaCO_3_) according to our results. 

Usually, lead degradation in a sea environment brings about the formation of several minerals, mainly *cerussite* (PbCO_3_), *anglesite* (PbSO_4_) and most rarely *cotunnite* (PbCl_2_) [10], while the presence of *galena* is typical of anaerobic or polluted sites. Considering the redox potentials for oxidation of lead to lead sulphate (−0.278 volts in normal seawater) and lead sulphide (−0.780 volts in 10^−6^ M sulphide) it is clear that the formation of PbS is thermodynamically favourable in anaerobic conditions. Furthermore, lead corrosion in anaerobic environments would be expected to be higher than in aerobic conditions, which can also favour the complete degradation of the lead, usually preserved by the formation of insoluble PbSO_4_, which soon forms a passivating surface film [38].

Wreck-wood decomposition involving the action of sulphate-reducing bacteria can occur in anaerobic conditions (SRBs), by producing sulphide ions which can react with the oxidised copper [39,40]. During this interesting biological-assisted corrosion process, bacteria participate in different steps of the corrosion process through their enzymatic systems. The hydrogenases are able to depolarise the metallic surface to solubilise the metal and the produced electrons move to sulphate which is reduced in sulphur and involves the dissolution of the metal. At the same time, the excretion of extracellular polymeric substances (EPS) improves the dissolution. The biofilm developed by D. desulphuricans at the metal surface accumulates with exposure time forming a poor protection patina [11]. Some examples are the sulphides are present in corroded bronze that came from two quite recent wrecks, *Wasa* (1628 in Stockholm Habour) [41] and *Mary Rose* (1545 near Portsmouth) [42], and from two Montefortino helmets [43].

## 4. Conclusions

In this paper, the results of the investigation of metal finds (nails and sheathings) belonging to the wreck of a Punic Ship exhibited at the Archeological Park of Lilybaeum of Marsala (Trapani, Italy) are reported. The results showed that the nails are made from unalloyed copper, because the tin was not identified, definitively answering the question of the archaeologists. The traces of arsenic and antimony are evidence of the metallurgic process based on the use of not completely roasted sulphide ores and/or rather poor refining practices. However, today no more traces of metals is observed, but the metal is converted into sulphides and sulphates. On the other hand, despite the total absence of metallic structure, the nails’ shape was maintained. This is due to the peculiar anaerobic mineralisation, which generates some layers with heterogeneous composition; in detail, the external layer contains *covellite* mixed with quartz coming from the seabed as interaction growth layer metal/environment, while the inner layer contains *covellite* and *chalcanthite*, indicating that sulphides are transformed under aerobic condition into the more stable sulphates. Even the lead of the sheathing was totally converted to lead sulphides and sulphates maintaining the shape of the nails but together with the formation of a well-defined layer of concretion mainly made of sand. 

For both kinds of metals, the presence of sulphides is an indication of an underwater anaerobic environment, as observed for the two Montefortino helmets, which are actually dislplayed at the same museum but recovered in a different sea area. 

This study highlights how the seabed condition can involve the formation of peculiar compounds by driving the mineralisation of the metal. The obtained information will support defining the strategy of conservation.

## 5. Methodology and Instrumentation

*p*XRF *spectra* were acquired in situ by using a Tracer III SD Bruker AXS portable spectrometer. The irradiation by a Rhodium Target X-ray tube operating at 40 kV and 11 µA and the detection of fluorescence X-rays by a 10 mm^2^ silicon drift X-Flash detector allows the detection of elements with atomic number Z > 11. A window of 3–4 mm in diameter determined the sampled area. Each spectrum was acquired for 30 s. Ar, Ni, Pd and Rh signals, due to the atmosphere and instrumental components, are also present in all spectra. The S1PXRF® software (Version 3.8.30) was used for data acquisition and spectral assignments. The fluorescence signal area was estimated once the de-convolution of the whole spectrum was performed by using the software ARTAX 7 (Version 7.4.6.1).

*Raman spectra* were collected in situ by a next-generation handheld Raman spectrometer (BRAVO), manufactured by BRUKER. BRAVO is equipped with two excitation lasers (DuoLaser™ Excitation) with wavelengths centred at 785 and 853 nm working together to mitigate the fluorescence phenomena and offering the highest sensitivity across the entire spectral range. The spectra were collected in the 300–3200 cm^−1^ range in automatic set-up mode. The spectra were acquired on the surface of the finds without any sample manipulation. 

*XRD patterns* were acquired by a Philips PW 1050/39 diffractometer on the powdered samples. The diffractometer operates in the Bragg–Brentano geometry using Ni-filtered Cu Kα radiation (λ = 1.54056 Å) in the 2θ range of 5–75° with a step of 0.05° and a time for steps of 5 sec. X’pert HighScore® Software (Version 2.0) was used for the identification of the crystalline phases.

*Digital optical microscopy* was performed by using a Digimicro Profi USB by 5.0 MPixel; several macro-photo were acquired in sequence and then merged together to get a high-resolution micrograph of the whole cross-sections.

*Polarised optical microscopy* was performed on the cross-sections by using a Zeiss Imager.A2m microscope operating in reflecting light, equipped with crossed polarisers. Photomicrographs were captured via a Photometrics AXIOCAM ICC1 digital camera mounted atop the microscope. Images were recorded at a magnification of 50x, both in dark and bright fields.

*SEM investigations* were performed on the cross-sections by using a Phenom Pro X, Phenom-World (The Netherlands) with an optical magnification range of 20–135x, an electron magnification range of 80–130,000x, maximal digital zoom of 12x, acceleration voltages of 15 kV and an energy-dispersive X-ray spectrometer (EDS) detector with a nominal resolution of 10 nm or less. The microscope was equipped with a temperature-controlled (25 °C) sample holder. The samples were positioned on an aluminium stub using adhesive carbon tape.

The *cross-sections* were prepared by embedding the collected micro-samples into epoxy resin and curing until solidification. The resin was then cut and polished to obtain the stratigraphy across the centre of the objects. 

## Data Availability

The datasets generated during and/or analysed during the current study are available from the corresponding author upon reasonable request.

## Supplementary Materials

The following supporting information can be downloaded at: https://www.mdpi.com/article/10.3390/molecules28041968/s1, Table S1: Net area percentage of the element’s peaks identified in the XRF spectra of the nails; Figure S1: Photo of a nail inserted in a wood fragment and two details of the surface; Figure S2: Photo of a nail and two details of the surface; Figure S3. Photo of the two twin nails and two details of the surface; Figure S4. Photo of some nails inserted in the wreek of the ship, analysed by portable XRF; Figure S5. Raman spectra of some nails. The spectra of sample 3_red and sample 3_white are acquired on the red and white areas reported in Figure S2. The spectrum of sample 1_white is acquired on the white area reported in Figure S4. Figure S6. EDS acquired on the spot indicated on SEM micrographs.

## Sample Availability

Samples are stored at the Archeological Park of Lilybaeum, Marsala (TP, Italy).
