# Supplementary material for: New Insight on Archaeological Metal Finds, Nails and Lead Sheathings of the Punic Ship from Battle of the Egadi Islands
Source: Molecules. 2023 Feb 19;28(4):1968. doi: 10.3390/molecules28041968 (PMC9968109; doi:10.3390/molecules28041968)
Supplement: Supplementary file 1 [file molecules-28-01968-s001.zip › molecules-2197434-supplementary.pdf]

molecules

MDPI

Support information

New Insight on Archaeological Metal Finds, Nails and Lead Sheathings of the Punic Ship from Battle of the Egadi Islands

Francesco Armetta [1], Rosina Celeste Ponterio [2], Ivana Pibiri [1] and Maria Luisa Saladino [1,*]

[1] Department Biological, Chemical and Pharmaceutical Science and Tecnology, University of Palermo Viale delle Scienze Bld. 17, 90128 Palermo, Italy
[2] CNR—Istitute for Chemical Physical Processes , Viale Ferdinando Stagno D'Alcontres 37, 98158 Messina, Italy
* Correspondence: marialuisa.saladino@unipa.it

Tables

Table S1. Net area percentage of the element's peaks identified in the XRF spectra of the nails.

	Area %						
	**As K12**	**Ca K12**	**Cu K12**	**Fe K12**	**Pb L1**	**S K12**	**Sr K12**
1_body	0.47	9.54	80.00	6.89	0.88	1.78	0.44
1_tip	0.32	2.55	93.34	3.06	0.17	0.45	0.10
1_head	0.16	1.83	95.20	1.77	0.30	0.71	0.04
2_tip	0.40	2.92	88.18	7.37	0.24	0.82	0.08
3_body white	0.82	46.82	45.79	0.63	4.14	1.41	0.39
3_body red	0.50	52.43	29.19	8.48	6.71	2.27	0.42
3_body	0.45	12.61	80.57	0.77	3.15	1.95	0.49
3_tip inner	1.35	24.11	68.96	0.54	2.79	1.51	0.74
3_tip	0.21	1.81	95.06	0.73	1.05	1.12	0.03
3_head	0.73	15.92	76.80	1.35	2.07	2.24	0.90
5_nail broken	0.44	11.13	77.86	4.98	1.32	4.04	0.24
5_tip nail	0.30	1.81	79.70	16.45	0.31	1.32	0.11
5_head nail	0.08	1.21	96.09	1.50	0.09	0.99	0.04
7_head	0.40	8.73	82.69	2.60	3.72	1.59	0.27
7_head broken	0.48	0.91	96.46	0.40	0.69	1.05	0.02
8_yellow	0.20	0.90	26.98	68.52	1.38	1.88	0.14
8_head	0.94	3.57	78.73	10.87	3.87	1.82	0.20
ship_1	0.12	1.51	93.71	3.32	0.25	1.04	0.05
ship_2	1.58	1.29	59.80	0.80	33.56	2.87	0.08
ship_3	0.48	3.09	94.20	1.17	0.67	0.30	0.09
ship_4	1.10	0.76	68.10	0.99	27.25	1.71	0.08
ship_5	0.88	0.62	74.31	0.97	21.83	1.32	0.08
ship_6	0.09	0.33	96.02	1.02	1.88	0.64	0.02
ship_7	0.20	0.30	94.31	1.80	2.64	0.71	0.03
ship_8	0.16	0.88	92.37	2.33	3.99	0.23	0.03
ship_9	0.14	3.69	92.12	2.27	0.97	0.63	0.17
ship_10	0.20	1.19	93.23	1.74	2.49	1.12	0.03
ship_11	0.31	0.22	95.85	0.73	2.19	0.69	0.01
ship_12	0.46	0.95	84.20	1.91	11.44	0.97	0.07
ship_13	0.24	2.96	91.15	2.28	2.04	1.30	0.04
ship_14	0.40	1.17	90.98	4.21	2.84	0.30	0.10

Figures

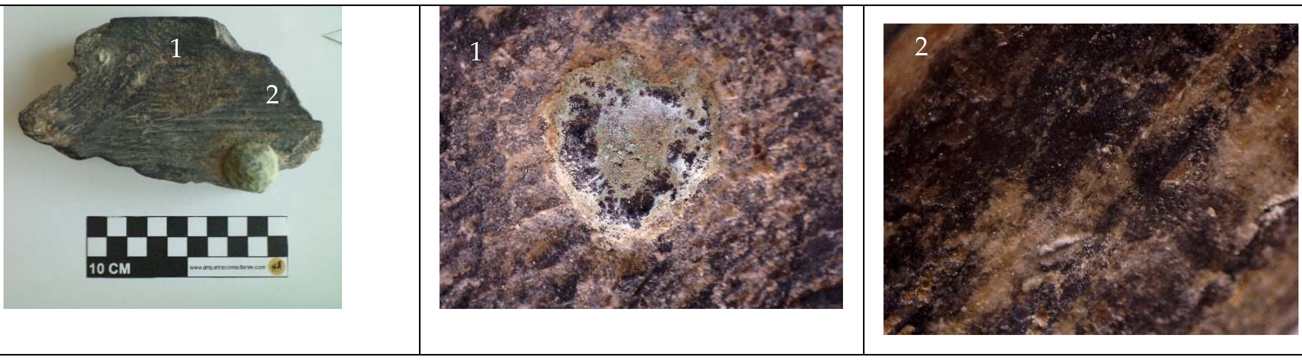

Figure S1. Photo of a nail inserted in a wood fragment and two details of the surface.

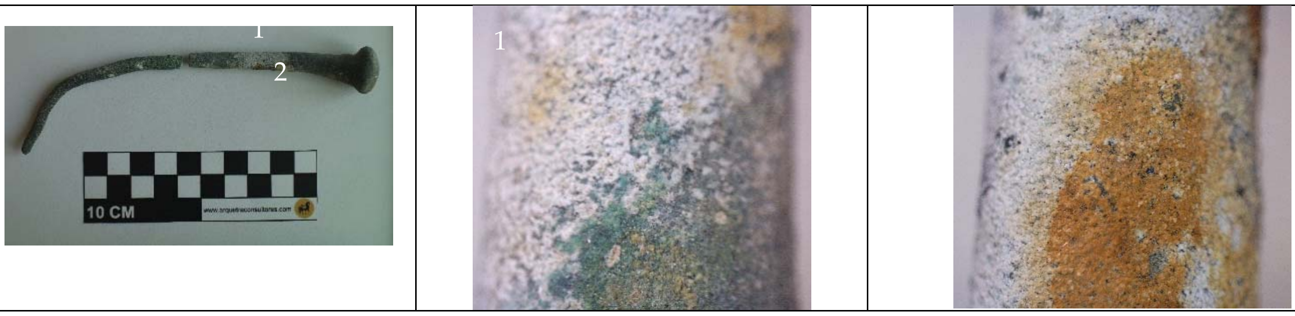

Figure S2. Photo of a nail and two details of the surface.

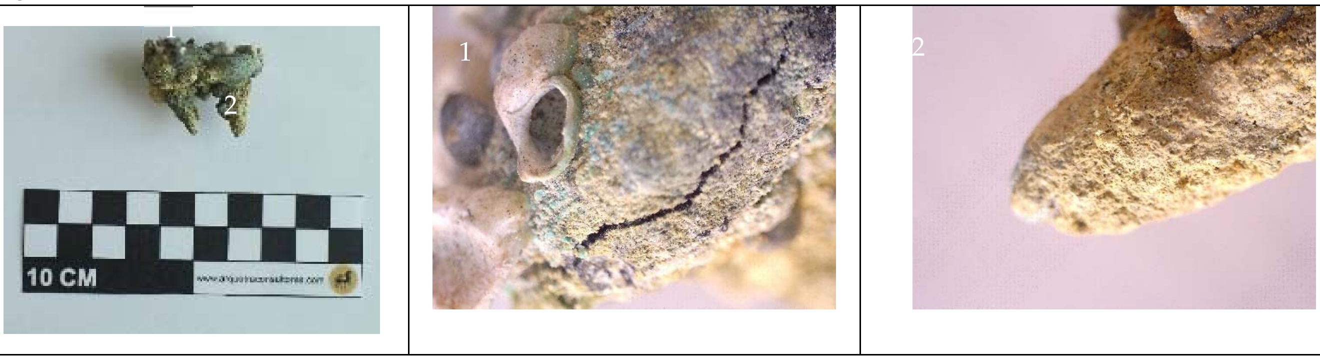

Figure S3. Photo of the two twin nails and two details of the surface.

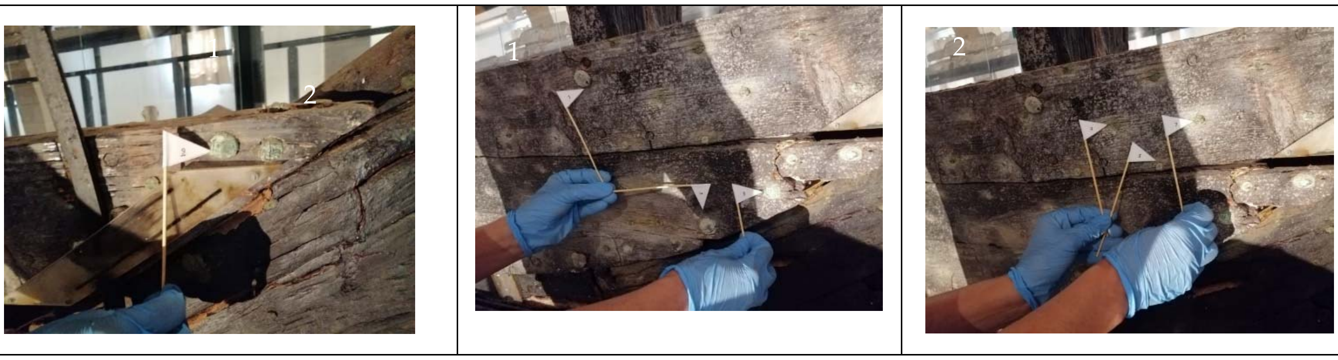

Figure S4. Photo of some nails inserted in the wreek of the ship, analysed by portable XRF.

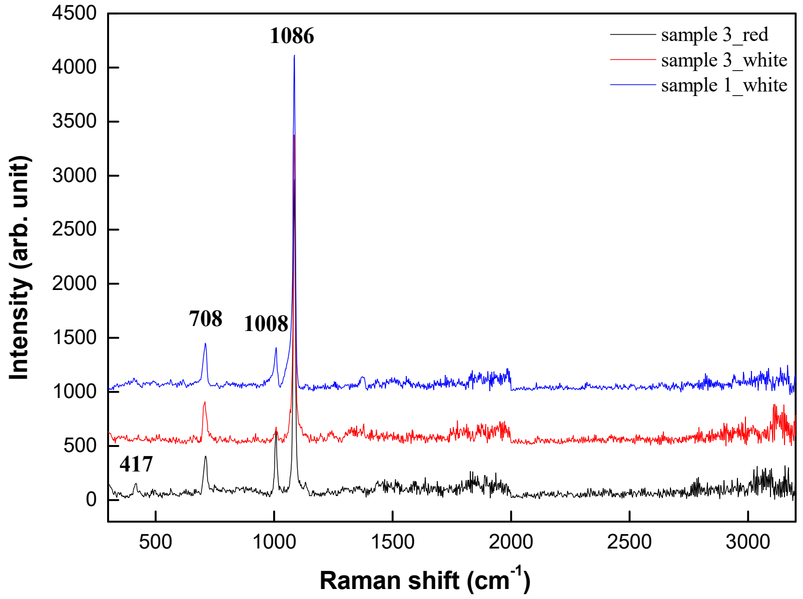

Figure S5. Raman spectra of some nails. The spectra of sample 3_red and sample 3_white are acquired on the red and white areas reported in Figure S2. The spectrum of sample 1_white is acquired on the white area reported in Figure S4.

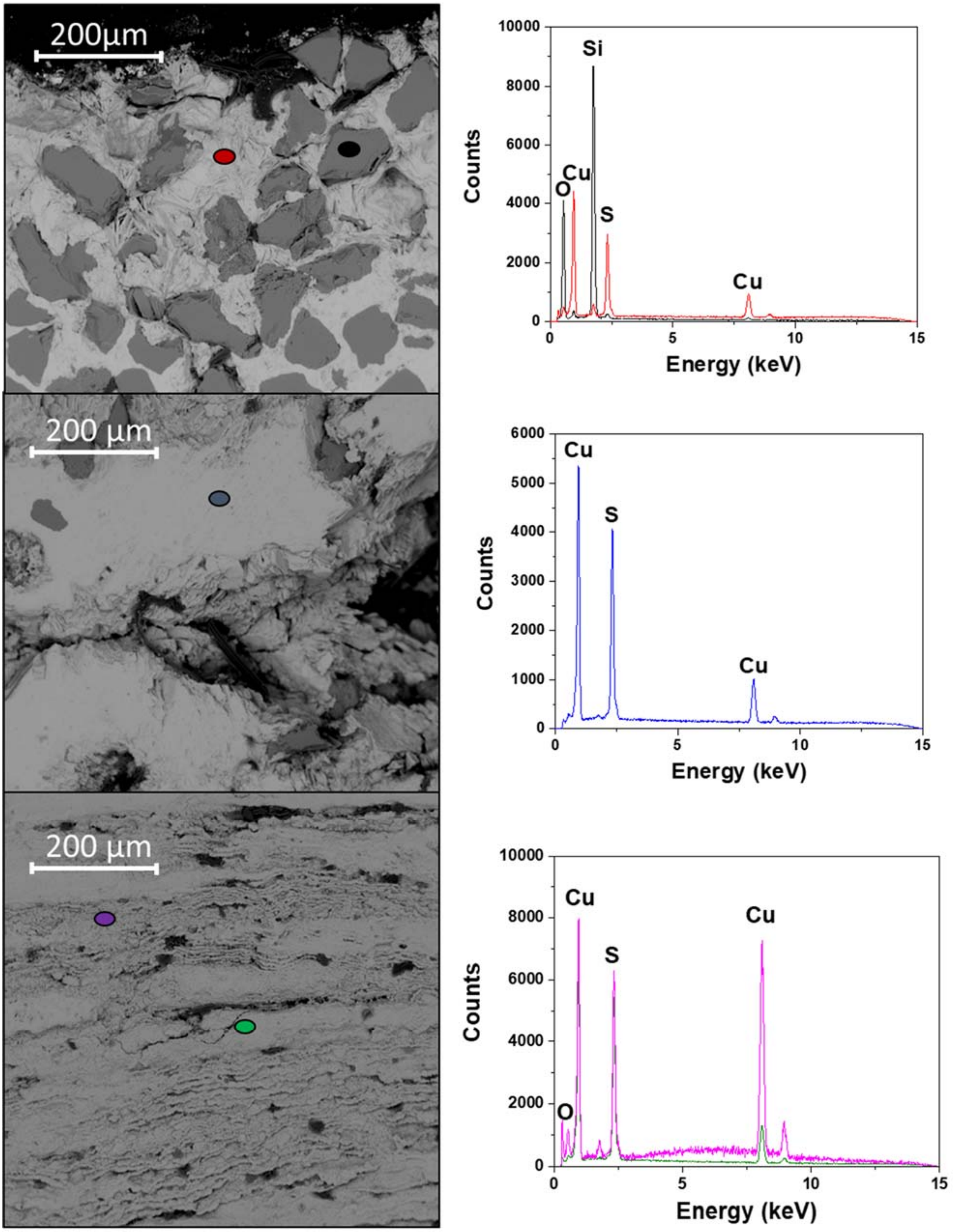

Figure S6. EDS acquired on the spot indicated on SEM micrographs.
